# Fast and Accurate Learning When Making Discrete Numerical Estimates

**DOI:** 10.1371/journal.pcbi.1004859

**Published:** 2016-04-12

**Authors:** Adam N. Sanborn, Ulrik R. Beierholm

**Affiliations:** 1 Department of Psychology, University of Warwick, Coventry, United Kingdom; 2 Department of Psychology, Durham University, Durham, United Kingdom; 3 Centre for Computational Neuroscience and Cognitive Robotics, University of Birmingham, United Kingdom; University of Minnesota, UNITED STATES

## Abstract

Many everyday estimation tasks have an inherently discrete nature, whether the task is counting objects (e.g., a number of paint buckets) or estimating discretized continuous variables (e.g., the number of paint buckets needed to paint a room). While Bayesian inference is often used for modeling estimates made along continuous scales, discrete numerical estimates have not received as much attention, despite their common everyday occurrence. Using two tasks, a numerosity task and an area estimation task, we invoke Bayesian decision theory to characterize how people learn discrete numerical distributions and make numerical estimates. Across three experiments with novel stimulus distributions we found that participants fell between two common decision functions for converting their uncertain representation into a response: drawing a sample from their posterior distribution and taking the maximum of their posterior distribution. While this was consistent with the decision function found in previous work using continuous estimation tasks, surprisingly the prior distributions learned by participants in our experiments were much more adaptive: When making continuous estimates, participants have required thousands of trials to learn bimodal priors, but in our tasks participants learned discrete bimodal and even discrete quadrimodal priors within a few hundred trials. This makes discrete numerical estimation tasks good testbeds for investigating how people learn and make estimates.

## Author Summary

Studies of human perception and decision making have traditionally focused on scenarios where participants have to make estimates about continuous variables. However discrete variables are also common in our environment, potentially requiring different theoretical models. We describe ways to model such scenarios within the statistical framework of Bayesian inference and explain how aspects of such models can be teased apart experimentally. Using two experimental setups, a numerosity task and an area estimation task, we show that human participants do indeed rely on combinations of specific model components. Specifically we show that human learning in discrete tasks can be surprisingly fast and that participants can use the learned information in a way that is either optimal or near-optimal.

## Introduction

People are often asked questions that require discrete numerical estimates. When judging from a glance “How many people are in the room?” or “How many dots are on a screen?” the quantity to estimate is discrete and any sensible answer must be a whole number. Discrete numerical estimates are also often required when the underlying quantity is continuous, very commonly when buying items. For example, painters often quickly assess a wall, whose area is a continuous quantity, and then buy a discrete number of paint cans; likewise at parties hosts may have to assess their guests’ hunger and order a discrete number of pizzas.

To understand how discrete numerical estimates are made, a formal framework is needed. Perhaps the most prevalent formal framework for characterizing decision making is Bayesian decision theory, which has provided a normative standard against which to measure behavior in economics [1–3], biology [4, 5] and for a wide variety of tasks in psychology such as categorization, memory, multi-sensory and sensorimotor integration, and reasoning [6–11]. Bayesian decision theory prescribes how to combine prior beliefs about states of the world, the likelihood that a state of the world generated an observation, and the decision function—the function which converts an uncertain representation into the response that maximizes expected reward. The interaction between these components is fixed in Bayesian decision theory, but the prior, likelihood, and decision function can each take many forms. This freedom allows Bayesian decision theory to correspond to a wide range of decision making behavior [12, 13], and in the right experimental design each component can be identified [14–17]. Identifying the prior characterizes how people represent past experience, identifying the likelihood indicates how people represent the evidential value of a new observation, and identifying the decision function characterizes how people convert uncertain beliefs into an estimate.

Despite the prevalence of real-world decisions requiring discrete numerical estimates, not much work has been done to characterize them using Bayesian decision theory to determine the priors, likelihoods, and decision functions that people use (cf. [18]). This stands in contrast to investigations of continuous estimates, such as pointing to a location, which have received much more attention [10, 15, 19–25]. In this paper we address this gap by characterizing how people make discrete numerical estimates. We first describe Bayesian decision theory and how continuous estimates have been characterized. Next we review previous research into discrete numerical estimation, finding it to be sparse but suggestive of differences in how continuous and discrete numerical estimates are made. We then present three new experiments that investigate discrete numerical estimates using bimodal training distributions, which allowed us to identify both the prior and decision function used. Our first experiment uses a numerosity task in which both the ground truth and response are discrete. In our second experiment, we generalize our results to a rectangle area estimation task in which the ground truth is continuous, but participants must give discrete estimates in whole square centimeters. Participants in the first two experiments learn bimodal priors quite quickly, so the third experiment investigates learning of more complex quadrimodal training distribution, which allows us to better distinguish how participants build their priors from experience. Finally we discuss our results, comparing continuous and discrete numerical estimation, exploring the implications for how people learn priors, and discussing how people convert uncertain beliefs into a numerical estimate.

### Characterizing continuous estimates

To characterize how people make continuous estimates, first we outline Bayesian decision theory, which prescribes how to maximize expected rewards. Bayesian decision theory is composed of three components that each need to be specified: the prior probability, the likelihood, and the decision function. There are a variety of distributions and functions that can be used for each component, but how the components are combined is fixed by the laws of probability [26].

The decision maker begins with a prior *P*(*S*), which gives the prior probability of each state of the world, *S*. For simplicity, we assume that the states of the world all are arranged along a single dimension and each state has a one-to-one mapping to a response. On each trial, the decision maker observes some data, *X*, that are noisy or ambiguous, and the likelihood, *P*(*X*|*S*), is the probability of the observed data given each of the possible states of the world. The prior and the likelihood are combined via Bayes’ rule to determine the posterior probability of the states of the world having observed the data,

P(S|X)∝P(X|S)P(S) (1)

where equality is achieved if the right-hand side is divided by *P*(*X*). In a sequential task, this posterior distribution is used as the prior distribution for the next trial, so the prior reflects a participant’s accumulated experience throughout the task.

The best estimate depends not only on what is believed to be true about the world; because *S* given *X* is uncertain, it also depends on what happens if an incorrect response is made. The dependence of rewards on the response is given by the loss function *L*(*R*; *S*), which captures the loss (negative reward) for making response *R* if the state of the world is *S*. The decision function, *D*_*L*_, then maps the posterior probabilities onto the response with the smallest expected loss

$$D_{L}\left(P\left(S|X\right)\right)=\underset{R}{arg min}\int_{S}L\left(R;S\right)P\left(S|X\right).$$ (2)

Continuous estimates have been modeled using a particular set of priors, likelihoods, and decision functions. *Likelihoods* are often assumed to be Gaussian because this density is a good match to the perceptual noise in many tasks, and participants have been shown to correctly adapt to the amount of noise in their perception. For example, in multi-sensory integration and sensorimotor tasks, the normative weight applied to each sensory cue depends on the variance of Gaussian-distributed perceptual noise, and participants’ weights come close to matching these normative weights [8, 10, 27].

For the *prior*, a standard choice for the training distribution in continuous estimation tasks is a Gaussian density because it makes the analysis analytically tractable: combining a Gaussian prior with a Gaussian likelihood results in a Gaussian posterior. However more flexible schemes for learning priors exist, and a common way to introduce flexibility is to use a non-parametric prior which grows in complexity as more data are observed. Kernel density estimation is a standard choice for building a non-parametric prior for continuous training data [28] and this kind of representation has been used in many models of human categorization [29, 30]. In kernel density estimation, the nonparametric prior is constructed from a weighted sum of component parametric densities, one for each previously observed data point. Mixture priors, which have also been used in models of categorization [7, 31, 32], provide another representation that allows more flexibility in the number of parametric components than kernel density estimation. This representation operates between the simple parametric and the kernel density cases, grouping similar data together into the same component, but allowing different components for data that are dissimilar.

In continuous estimation tasks, participants learn Gaussian and other unimodal training distributions quickly: in various continuous estimation tasks, unimodal training distributions have been learned in hundreds of trials [10, 19, 21, 23]. Participants can also learn bimodal training distributions, demonstrating that they do not use just simple parametric priors, but they are slower to do so. Experimenters have had to train participants on bimodal distributions for thousands of trials [10, 22], because fewer training trials do not result in clear evidence of learning [19]. Participants were able to use bimodal distributions when presented with an explicit summary, and this work also showed participants are better described as using a mixture prior rather than kernel density estimate with a very narrow kernel [15].

For the *decision function* in continuous estimation, a small number of simple functions have been considered, each of which can be motivated by one or more loss functions. An all-or-none loss function leads to choosing the response with the highest posterior probability, a quadratic loss function leads to taking the mean of the posterior, and a linear loss function yields the posterior median as the best response. A fourth decision function, drawing a sample from the posterior, requires a more complex motivation. One route is to speculate that participants assume that the computer is adaptively responding to their input in a competitive fashion, so that a stochastic decision function can increase their expected reward [33, 34]. Another is to assume that participants are maximizing expected reward subject to particular computational costs: if participants draw samples from the posterior and these samples require time or effort to generate, it can be better to make a quick and less accurate decision rather than a slow and effortful accumulation of enough samples to calculate the maximum of the posterior [35, 36].

Comparison of these decision functions in continuous estimation has yielded task-dependent conclusions. Some researchers have found evidence for the mean decision function, finding that participants used a loss function that was quadratic near the correct value but more linear far from the correct value, giving it robustness to outliers [24]. Other work has found evidence for a mean decision function despite feedback which did not encourage this decision function [23], but a later analysis showed that this particular task does not discriminate well between decision functions [14].

Recent research has incentivized the max decision function and then investigated the decision function actually used. Work using Gaussian priors found that instead of the max, participants were performing an interpolation between drawing a single sample and taking the max of the posterior [20]. There are various mechanisms that could produce this interpolation: participants could be drawing a number of samples from the posterior distribution and taking the mean of these samples as their estimate, they could be raising the posterior to a power greater than one and then sampling their estimate from this exponentiated posterior distribution, or perhaps they combine the two by taking the mean of a number of samples from an exponentiated posterior. While these explanations are indistinguishable for Gaussian posteriors [20], work using a bimodal training distribution has successfully tested two of these possibilities, the mean of a number of samples versus a sample from an exponentiated posterior, and found that participants were drawing a single sample from an exponentiated posterior distribution [15].

### Characterizing discrete numerical estimates

Though researchers have occasionally investigated discrete numerical estimation, it has not received much attention, possibly because it has been viewed as no different from continuous estimation. However if we look at the work that has been done, there are suggestions that people make these two types of estimates differently. Bayesian decision theory is useful here for cataloguing the similarities and differences.

The *likelihood* in discrete numerical estimation is similar to that found in continuous estimation. As in continuous tasks, participants find it more difficult to discriminate stimuli that are closer physically even though they are naturally discrete. Indeed, in perceptual numerosity tasks, discrimination performance nearly follows Weber’s law, implying that the standard deviation of the perceptual noise distribution is proportional to its mean [37], which has been modeled as Gaussian noise on the log-transformed numbers [38–41]. Like in continuous tasks, participants appear to use knowledge of their own perceptual noise to set their likelihood in numerosity tasks [38], a point we also address in the S1 Methods.

Investigations into the *priors* used in discrete numerical estimation have shown that participants can learn unimodal distributions of stimuli quickly, as in continuous estimation. Participants are able to reconstruct the frequency of events from unimodal distributions from just a few trials [42] and their estimations of new events quickly show an influence of the mean in a changing sequence of numbers [43]. In the similar task of absolute identification, in which participants are asked to identify a series of perceptual stimuli with numerical labels [44], participants are also influenced by unimodal distributions of stimuli [45].

However, tasks training bimodal prior distributions point to potential differences between continuous and discrete numerical estimation. The first potential difference is in the speed of learning bimodal priors. In one task, participants asked to reconstruct bimodal prior distributions were able to do so within a few hundred training trials [18], and in another participants could do so for some bimodal distributions after only 12 trials [46]. Though this suggests that participants have a speed advantage in learning priors for discrete numerical estimates, these priors were assessed through reconstruction and it needs to be established whether the same priors are used in estimation.

A potential difference in the *decision function* was also found in [18], in an experiment in which participants were asked to estimate the revenues associated with trained and novel company names. Participants’ estimates for novel companies were either at the lower edge of the range of trained revenues or were in the middle of the range, results which were modeled as drawing a set of samples from the prior combined with a mixture of two decision functions. One decision function was to use the lowest sample in the set as the estimate (because unknown companies are likely to have low revenue), and the other was to take the mean of the set of samples as the estimate [18]. The use of the mean of a small number of samples as the decision function contrasts with the exponentiated posterior supported by work in continuous estimation, and this is another potential difference. However, revenue estimation is very different from the perceptual tasks used in continuous estimation, so it would help to investigate the decision function in a perceptual discrete numerical estimation task.

Here we investigate these potential differences in two different discrete numerical estimation tasks: estimating the number of dots on a screen and estimating the area of a rectangle. We are particularly interested in whether participants can quickly learn complex multimodal prior distributions and what decision function they use to make their estimates. In exploring this, we go further than previous studies by investigating whether participants use a kernel density estimate, a mixture model, or a categorical distribution as a prior. Through both combinatorial model comparison and fitting of nested models we examine the decision function, investigating whether the mean, max, the mean of a number of samples from the posterior, a sample from an exponentiated version of the posterior distribution, or perhaps a more complex decision function best explains participants’ estimates. We compare our findings to the results from continuous estimation in the discussion as well as explore the implications for what priors people can learn and what decision functions they use.

## Results

### Experiment 1

**Fig 1:**
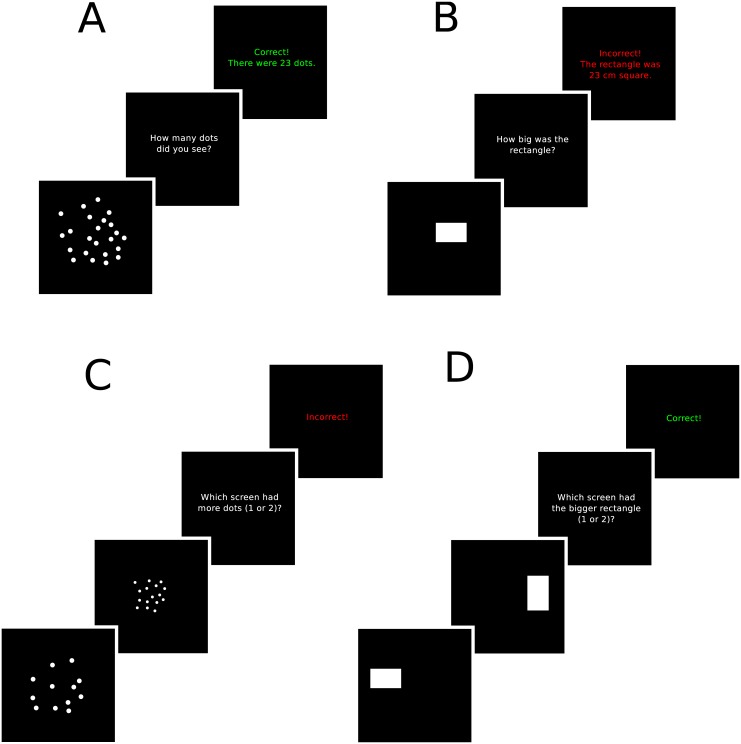
Illustrations of the tasks used in the experiments. A) Estimation trials for dots. B) Estimation trials for rectangle area. C) Discrimination trials for dots. D) Discrimination trials for rectangle area.

**Fig 2:**
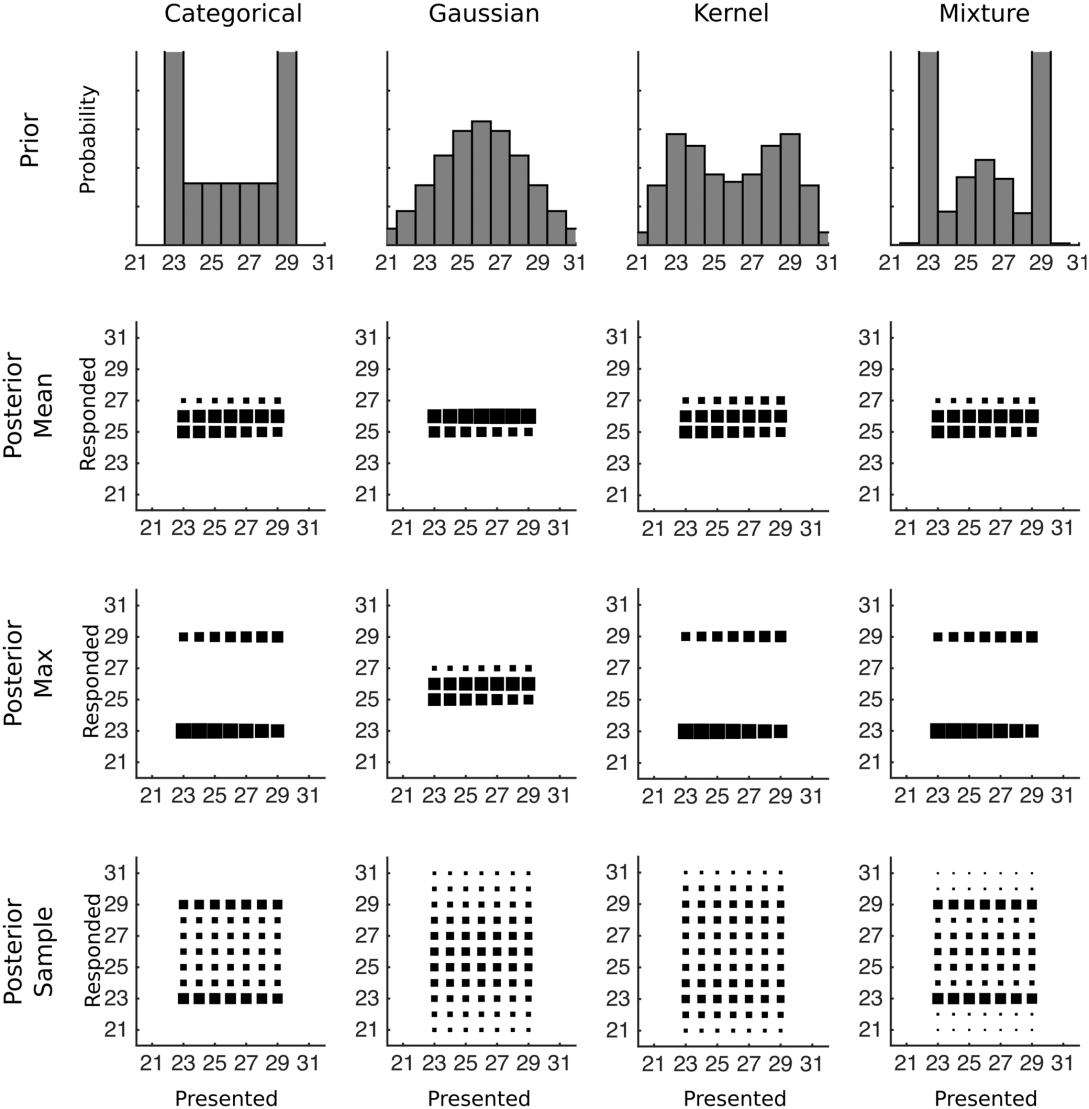
The predicted data for various combinations of prior distribution and decision function for Experiments 1 and 2. The first row shows how a strongly bimodal distribution would be represented by each type of prior after training, with the categorical distribution reflecting the true prior distribution. The remaining rows of plots each show conditional response distributions (CRDs), in which the area of each square represents the expected response probability given a particular presented number of dots. The prior in the first row is combined with each row’s decision function to produce the CRDs.

To characterize how participants make discrete numerical responses, we ran a new experiment on numerosity estimation in which participants were trained on a bimodal distribution. Participants were asked to estimate the number of dots that briefly appeared on a screen in a series of trials, receiving feedback about whether they were correct and what the correct answer was after each trial as shown in Fig 1A. They were not told anything about which numbers to expect in addition to the feedback. On each trial, the number of dots on the screen was drawn from a sharp bimodal distribution with two peaks on either side of a region of lower probability values (e.g., the distribution shown in the top left corner of Fig 2). A sharp bimodal distribution allows us to better identify the prior used. If participants are not generalizing beyond the numbers that were given as feedback, then their prior should eventually match the training distribution and they will not respond outside the range of the stimuli. However, if participants are using a parametric or kernel density prior distribution, then the prior distribution will have some spillover outside the range of stimuli, and participants will respond outside this range even after hundreds of training trials. Using a mixture prior will also result in responses outside the range, but they will likely be fewer in number. Examples of these four possibilities are shown in the top row of Fig 2.

Before and after the main task, we included a separate discrimination task that allowed us to characterize the likelihood distribution for each participant. This removed a degree of freedom from the process of characterizing the prior and decision function. The noise in numerosity judgments is well-known to follow Weber’s law with a standard deviation proportional to the mean [37], and has been modeled in past research as a lognormal distribution [38–41]. We assumed that the likelihood distribution was accurately calibrated and thus equivalent to the noise distribution. The scale of the lognormal distribution *σ* (i.e., the standard deviation of the natural logarithm of a lognormally distributed variable), which can be determined from the Weber fraction *w* using the formula $\sigma =\sqrt{\log(w^{2}+1)}$, was estimated in a discrimination task in which participants were asked which of two screens contained more dots (shown in Fig 1C). Participants’ discrimination judgments were well fit with a standard deviation that ranged from 0.18 to 0.53, with a median of 0.22. These estimates are in reasonable agreement with previous research that found for numerosity estimates that discriminability was equivalent to *σ* ≈ 0.16 [37, 47].

After fixing the lognormal standard deviation, we can make predictions for the responses after the training distribution has been learned for each pairing of prior and decision function. Using the median estimate of *σ* = 0.22, predictions from pairings of possible priors and decision functions are shown in Fig 2 in the form of conditional response distributions (CRDs): for trials on which a particular number of dots are presented, each panel shows the distribution over the responses expected by the combination of prior and decision function. Two qualitative features stand out in these plots. The first is the number of modes in each CRD. If the mean decision function is used or if the prior is a Gaussian distribution then the CRD will be unimodal, otherwise it will be bimodal. The second qualitative feature is whether responses occur outside the range of values that was presented. Responses will always be within the range of presented values for the categorical prior, while for other priors responses can occur outside of the range if participants sample from the posterior distribution.

**Fig 3:**
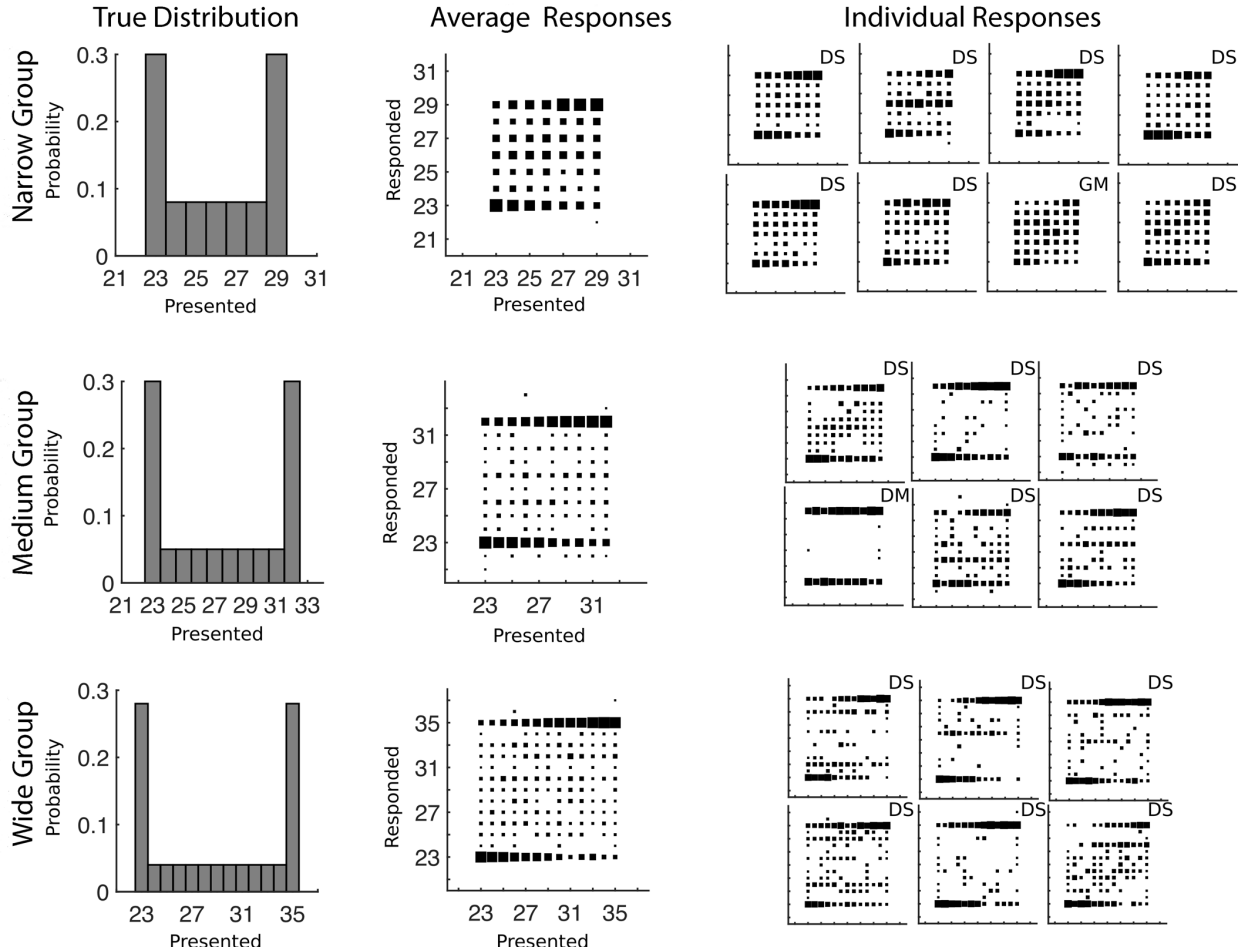
Training distributions and conditional response distributions for each group of participants after 300 trials in Experiment 1. Each group of participants is in a separate row. The left column of plots shows the probability with which each number was presented to participants. The middle column shows aggregate conditional response distributions with the area of each square representing the relative frequency of making a particular response given a particular presented stimulus. The right column shows the conditional response distributions for each participant. The letters D and G mark participants who are using a categorical (Dirichlet prior) or Gaussian kernel prior respectively and the letters A, M, and S mark the participants who were characterized as using the mean (Average), Max, or Sampling decision functions respectively.

In the main task, participants were assigned to one of three groups, with each group participating in a series of trials in which the sharp bimodal prior distribution covered a larger or smaller range. This was done to ensure that the results were not strongly dependent on the distance between peaks or on the particular numbers assigned to the modes of the distributions. The average and individual CRDs for each of the three groups are shown in Fig 3, along with the distribution of the training trials given to participants. In order to plot stable performance, the first 300 trials were not included in these plots. The empirical CRDs for each group show a strong bimodality, implying that neither the mean decision function nor the Gaussian prior characterize human data. Responses are not made only at the modes of the training distribution, however, as a large number of responses are found between the two peaks. These middle responses are evidence against the max decision rule (which would be very unlikely to result in an intermediate response), so from qualitative inspection posterior sampling is left as the best characterization of the decision function.

Further inspection of the average data shows very few responses outside of the range of presented values: the narrow group shows no such responses, and the medium and wide groups show few responses of these types. For the medium and wide groups, the responses outside the range of presented values only appear for a small subset of the participants: the third and fifth participant in the medium group, and the third and fourth participant in the wide group. The lack of responses outside of the range of presented values, combined with the identification of participants’ decision function as consistent with posterior sampling, implicates the use of a categorical prior, though this is not easy to distinguish from a mixture prior, as shown in Fig 2.

We fit a set of computational models (see Methods; Model comparison) to provide quantitative evidence that individual participants were using categorical priors and sampling from the posterior. Each model was fit to all of the trials and the prior was updated after each instance of feedback was given. We specifically tested the combinations of prior updating (categorical (Dirichlet) or Gaussian kernel) and decision functions (mean (Average), Max or Sample). We performed a model comparison using the Bayes Information Criterion that adjusts the fit of the model with a penalty for complexity [48]. Eighteen of the twenty participants in this experiment were best described by the categorical prior and a decision function that drew a single sample from the posterior. For the remaining two participants, one was best described by a Gaussian kernel and a max decision function and the other by a categorical prior and a max decision function. The best models for each participant are indicated in Fig 3 and the BIC values transformed into approximate posterior probabilities are shown in the S1 Methods.

To allow for a wider range of possible behaviors, we also fit computational models that allowed for “trembling hand” noise and models that allowed the posterior distribution to be raised to a power before the decision function was applied (see Methods). Once we included this set of models, we found that nineteen of the twenty participants were best described by raising the posterior distribution to an exponent larger than one before the decision function was applied, while the remaining participant was best described with the original model (implying an exponent of one). Once the posterior distribution was raised to a power, behavior was best described as a single sample for ten participants and as the mean of the exponentiated posterior for nine participants. This generalization elaborates on what was found with the first set of computational models: exponentiating the posterior means that participants lie between sampling and the max decision function, and the individual differences in using a single sample or the mean reflect individual differences in the amount of stochasticity in the estimates and in the tendency to sometimes respond near the middle of the presented range of stimuli.

A final generalization was to fit a ‘super-model’ to each participant’s data (see Methods) that allows us to further investigate the individual differences in stochasticity that participants have in their estimates by quantifying the number of samples they use. The individual best fits and an exercise showing that these parameters are identifiable are given in the S1 Methods. Reinforcing the model comparison above, nineteen of the twenty participants used an exponentiated posterior distribution to make their decisions: the exponent was well above 1.0 for all but one participant. This one participant was best fit by a single sample, so there was no evidence that any participants were taking the mean of a small number of samples from the untransformed posterior, instead participants were sampling from an interpolation between the posterior and a distribution that was entirely on the max of the posterior. This analysis allows us to further investigate those participants found to be using the mean of an exponentiated posterior. Half of these participants were best fit by taking the mean of between 2–4 samples, while the remainder were taking the mean of a larger number (i.e., about 30) samples.

In summary, this experiment established that the great majority of participants could learn essentially categorical priors when using discrete numerical responses and tended to respond using a decision function that was either a single sample or the average of multiple samples drawn from an exponentiated posterior distribution. A question then arises about the generality of the results. Are people using a categorical prior distribution because the number of dots is necessarily a discrete quantity? Or are they using a categorical prior distribution because of the discreteness of the responses?

### Experiment 2

To test whether the results of Experiment 1 were driven by the discreteness of dots or the discreteness of the response, we ran essentially the same experiment, but instead of asking participants to estimate the numbers of dots we asked them to estimate the area of rectangles. Like in the example of buying paint to cover a wall, rectangle area is a continuous quantity but we forced participants to make discrete responses: they were required to estimate the area of rectangles in whole square centimeters.

**Fig 4:**
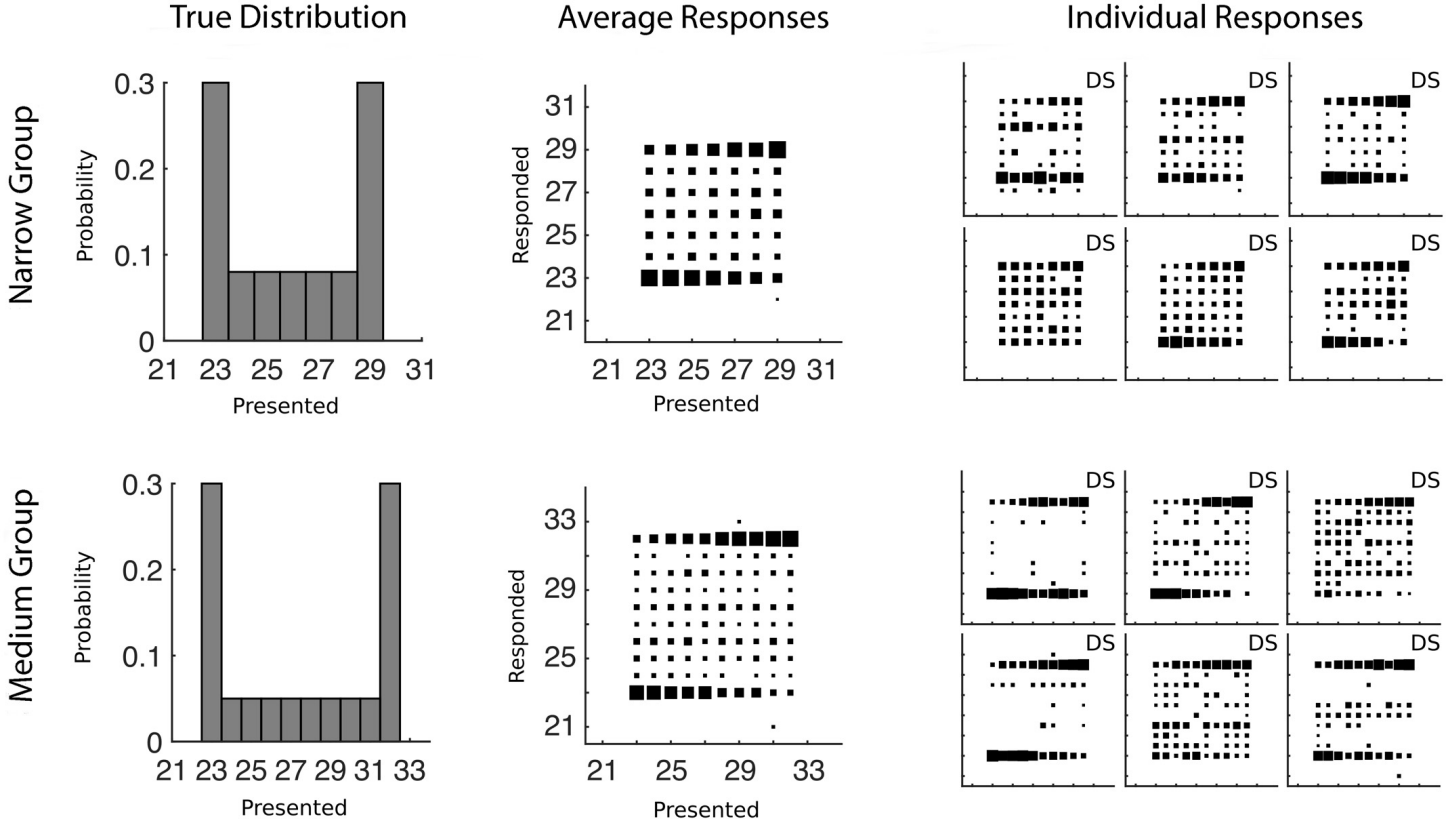
Training distributions and conditional response distributions for each group of participants after 300 trials in Experiment 2 (estimates of the area of a rectangle). Each group of participants is in a separate row. The left column of plots shows the probability with which each number was presented to participants. The middle column shows aggregate conditional response distributions with the area of each square representing the relative frequency of making a particular response given a particular presented stimulus. The right column shows the conditional response distributions for each participant. The letters D and G mark participants who are using a categorical (Dirichlet prior) or Gaussian kernel prior respectively and the letters A, M, and S mark the participants who were characterized as using the mean (Average), Max, or Sampling decision functions respectively.

Two groups of participants were run in this experiment and the results are shown in Fig 4. When we fit their discrimination data, we found a median of *σ* = 0.41. As in Experiment 1, the average results in the estimation task show a bimodal distribution. Responses often fall in the middle but hardly ever fall outside the range of presented values. This pattern again is qualitatively most consistent with a categorical prior and a decision function that draws a single sample from the posterior.

We used the same analysis approach of successive generalization with the same models as we used in Experiment 1, with all individual results given in the S1 Methods. In the simplest model comparison, we found that every participant was best described by a categorical prior distribution and a decision function that was a single sample from the posterior. This result is given next to each individual in Fig 4. When we generalized the comparison to allow the posterior distribution to be raised to a power before the decision function was applied, we found that every participant was better described by exponentiating their posterior distribution, bringing it closer to a distribution that was entirely on the max. As in Experiment 1, half of participants were best described by taking a sample from this exponentiated posterior, while the other half were best described by taking the mean of the exponentiated posterior reflecting a tendency to sometimes respond near the middle of the presented range of stimuli. The more general ‘super-model’ analysis provided more detail on how many samples were being taken from the exponentiated posterior, and thus the amount of stochasticity in the estimates. All of the participants were best fit by taking the mean of between 3 and 100 samples from an exponentiated posterior distribution, with the participants best described as taking the mean of the exponentiated posterior tending to be on the higher end of this range.

In both Experiments 1 and 2 participants used near-categorical prior distributions and either take a single or multiple samples from an exponentiated posterior when they are asked to make discrete responses, regardless of whether the underlying quantity was discrete or continuous. They clearly were able to learn a bimodal distribution surprisingly quickly, so these results lead to the question of how flexible this representation is. As shown in Fig 2 a mixture of Gaussians prior can quite closely imitate the categorical prior that was best supported by the data. As the mixture model interpolates between a Gaussian prior and kernel density estimation, it is difficult to provide evidence against this model. More generally, allowing mixtures of other types of distributions, such as uniform distributions, makes the problem even more difficult. In order to test whether participants were using mixture models, Experiment 3 investigates whether participants can learn a more complex prior distribution.

### Experiment 3

**Fig 5:**
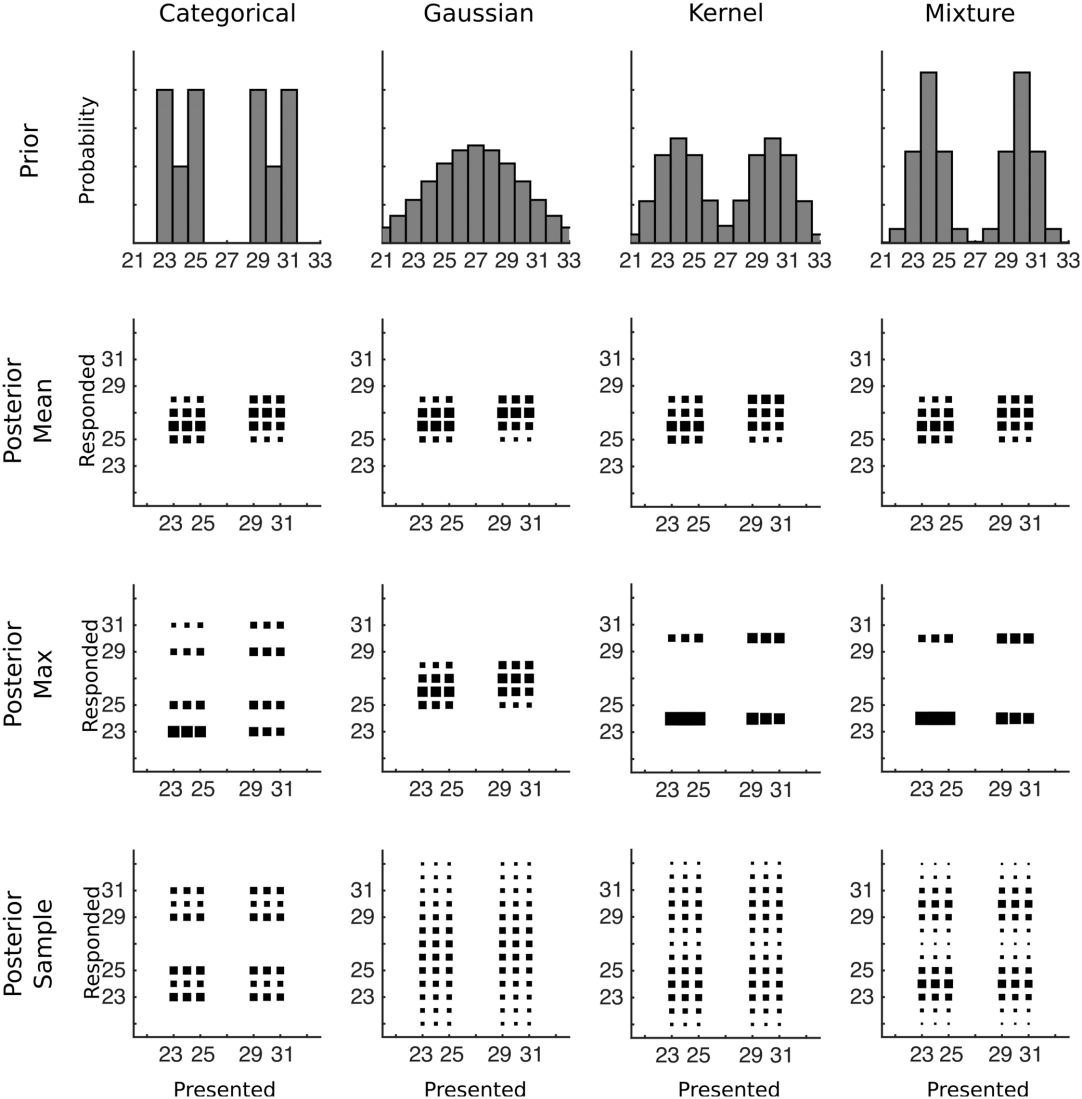
The predicted data for various combinations of prior distribution and decision function for Experiment 3. The first row shows how a strongly bimodal distribution would be represented by each type of prior after training, with the categorical distribution reflecting the true prior distribution. The remaining rows of plots each show conditional response distributions (CRDs), in which the area of each square represents the expected response probability given a particular presented number of dots. The prior in the first row is combined with each row’s decision function to produce the CRDs.

In order to further investigate how complex a prior distribution participants can learn within a few hundred trials, participants in this experiment were trained on a quadrimodal prior distribution. As shown in Fig 5, this distribution was designed to test whether participants were using simple mixture models. If participants are assigning all of the trials with 23–25 dots to one mixture component and all of the trials with 29–31 dots to a separate mixture component, then the predictions of the categorical prior and the mixture prior are clearly distinguishable: the mixture model always predicts a peak in response frequency at numbers 24 and 30, while the categorical prior distribution predicts that these numbers will be selected less often than the peaks. These same predictions would also be made if participants are using other distributions in a mixture model, such as uniform distributions, with the same assignments also leads to the prediction that numbers 24 and 30 will be selected at least as often as the other presented numbers.

**Fig 6:**
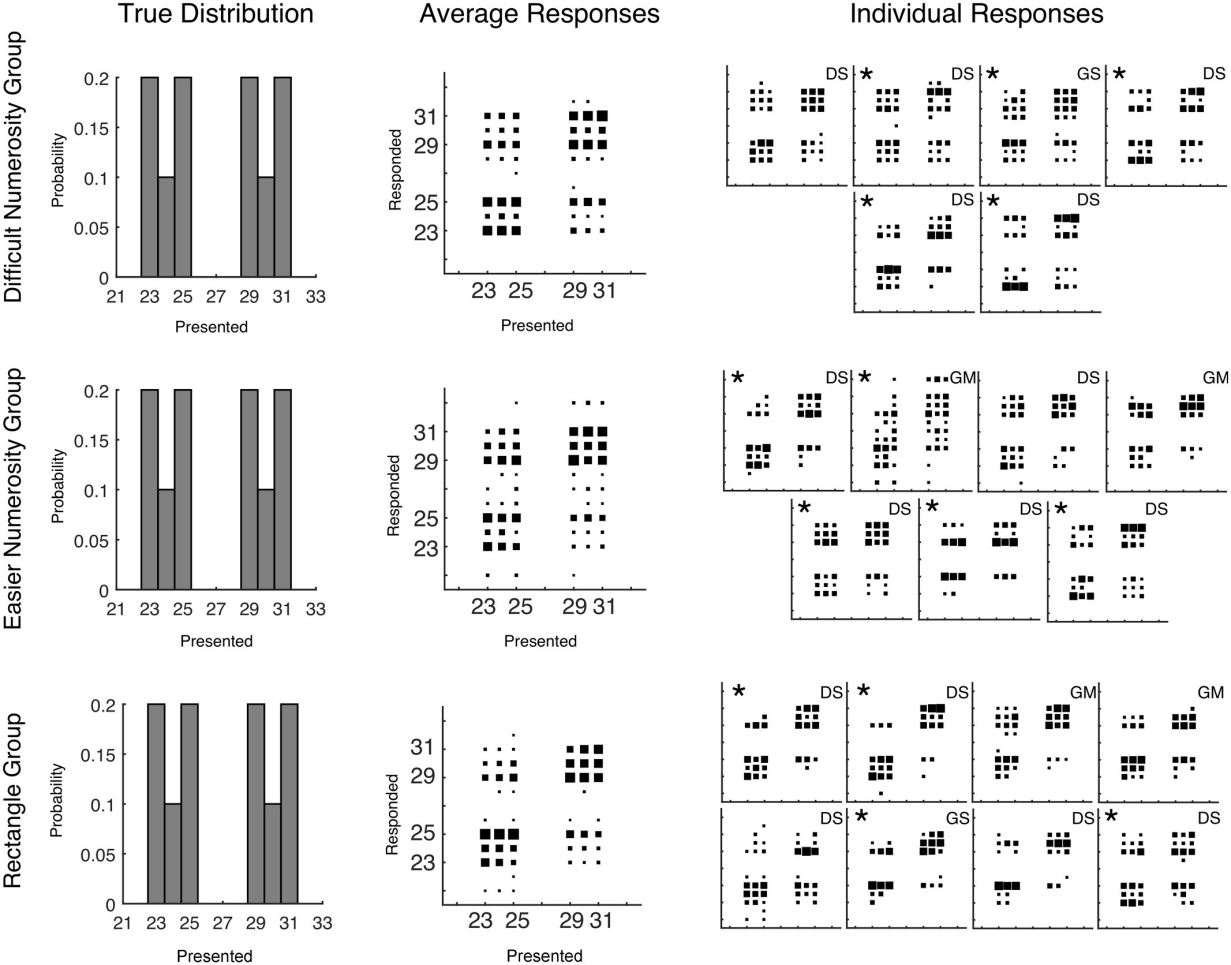
Training distributions and conditional response distributions for each group of participants after 300 trials in Experiment 3. Each group of participants is in a separate row. The left column of plots shows the probability with which each number was presented to participants. The middle column shows aggregate conditional response distributions with the area of each square representing the relative frequency of making a particular response given a particular presented stimulus. The right column shows the conditional response distributions for each participant. The stars mark participants that made significantly fewer responses to lower (but non-zero) probability numbers than higher probability numbers. The letters D and G mark participants who are using a categorical (Dirichlet prior) or Gaussian kernel prior respectively and the letters A, M, and S mark the participants who were characterized as using the mean (Average), Max, or Sampling decision functions respectively.

Three groups of participants were run in this experiment: one group completed a perceptually easier numerosity task, one group a perceptually more difficult numerosity task, and one group completed a rectangle task. When we fit the discrimination data, we found medians of *σ* = 0.19, *σ* = 0.14, and *σ* = 0.14 for the Difficult Numerosity, Easier Numerosity, and Rectangle groups respectively (first estimation task, see Methods) and used the latter value for generating Fig 5. The mean results for each of the groups are shown in Fig 6 and the qualitative results in this experiment again look like a combination of a categorical prior with sampling from the posterior distribution.

We ran the same analysis as was done for Experiments 1 and 2 (model comparison and fitting parameters of the ‘super-model’ with individual results given in the S1 Methods) to determine which model explained participants responses best. Using the simplest model comparison, 15 of 21 participants were best described by a categorical prior and by sampling their estimates from the posterior. The remaining six participants were better described by using a Gaussian kernel prior, with four of them taking the max of the posterior and two sampling. The correspondence of these results to the individual data is shown in Fig 6. The less restrictive model comparison again showed that a categorical prior and a single sample from an exponentiated posterior distribution was the best description of the largest number of participants (12 of 21). The other participants were best fit by a variety of models. For the ‘super-model’ analysis, which allows us to better investigate the stochasticity in the decision function, 11 out of the 21 participants drew a single sample from a posterior distribution that had an exponent clearly above 1.0, while the remainder either drew a single sample from a posterior distribution with an exponent not much different from 1.0 or took the mean of a larger number of samples from an exponentiated posterior distribution. No participants were best fit by averaging multiple samples from the unexponentiated posterior distribution.

To test whether participants were using a simple mixture model which assigned the trials with dots 23–25 to one mixture component and trials with dots 29–31 to separate mixture component, we looked at whether participants produced fewer responses of 24 and 30 compared to the peaks of the distribution. If participants were equally likely to respond with any of the presented numbers (after trial 300 and ignoring what the actual presented value was), then participants should have picked numbers 24 or 30 on at least 1/3 of trials. Using 1/3 of trials as a null hypothesis we ran binomial tests to determine if the actual number of responses was significantly lower than this value for each participant. Overall, 14 of 21 participants produced significantly fewer responses than the null hypothesis predicted (*p* < 0.05). The participant showing significant differences are marked with stars in Fig 6. Clearly a number of participants were not using this simple mixture model as their prior distribution.

Mixture models that are closer to the categorical prior are harder to rule out. For example, mixture model components might consist of separate components for every number, except for a single pair of numbers that are represented with the same component. For the prior distribution trained in this experiment, this would be a mixture model consisting of five component densities to represent the six presented responses. We simulated how often responses just outside the presented range would appear if there were separate mixture components for every number except for one single pair of adjacent numbers for a variety of choices of the adjacent pairs and values of *σ* and found that participants would be expected to produce a response just outside the range on perhaps as few as 0.6% of trials. This low rate means that it is not possible to say that any individual participant produced significantly fewer responses: the probability of producing zero of these responses on 200 trials assuming a 0.6% probability is 0.3. However, we do note that 11 of 21 participants did not produce any of these just-outside-the-range responses (and four additional participants produced only one). If all participants were consistently grouping adjacent numbers together the probability of observing this many participants with zero responses of this type is low (*p* = .027).

Overall, Experiment 3 demonstrates that participants are accurately learning very complex quadrimodal prior distribution within a few hundred trials. The complexity of the prior learned allowed us to even rule out for most participants a simple mixture model that could have explained behavior in the first two experiments.

## Discussion

In the preceding pages we have characterized the components necessary for optimal Bayesian decision making with discrete numerical stimuli and explained how model comparison and model fitting allows us to tease apart these components given the right experimental setup.

The results from three experiments show that most participants were better described by a perfect match to the training distribution, rather than a parametric distribution, a kernel density estimate, or some forms of mixture model. Generally speaking participants were best characterized as raising their posterior distribution to a power, which interpolates between the original posterior distribution and a distribution entirely on the max on the posterior. Using this transformed distribution, participants’ estimates were best described as averaging one or more of samples from an exponentiated posterior distribution, with the number of samples reflecting individual differences in the stochasticity of their estimates and in the probability of responding near the middle of the presented range of stimuli. As participants tended to either draw a single sample or use a large exponent to transform their posterior distribution, this ruled out the mean or the mean of a number of samples as viable decision functions. Experiments 1 and 2 established this pattern across both the numerosity and area estimation tasks, pointing toward the discreteness of the response rather than the discreteness of the stimuli as the driver of this behavior. Experiment 3 expanded this to even more complicated stimulus distributions, providing evidence against other mechanisms for updating the prior. We now compare our results to continuous and discrete estimation in previous experiments, and discuss the conclusions we can draw about priors and decision functions.

### Comparing continuous and discrete numerical estimation

Previous investigations had suggested that the decision function used in discrete numerical estimation might be different from that used in continuous estimation tasks. In tasks in which the max decision function was incentivized, work with continuous estimation tasks has shown that participants exponentiated the posterior distribution and drew a sample from this exponentiated distribution [15, 20]. This result contrasts with the findings from discrete numerical tasks showing that even when incentivized to use the max rule, participants still appeared to use the mean of a small number of samples [18].

Our results for discrete numerical estimation were different. We encouraged the max decision rule (by giving participants feedback of ‘correct’ or ‘incorrect’), and we found that participants were using an exponentiated version of the posterior distribution. Our ‘super-model’ analysis allowed for both exponentiation of the posterior and for the mean to be taken of a number of samples, and we found strong evidence for exponentiation in a large majority of participants and individual differences in whether a single or multiple samples were used. Despite the individual differences, no participants were best fit by the mean of a small number of samples from the untransformed posterior.

The divergent results between our task and the revenue estimation task of [18] need explanation. One potential key difference is that the likelihood was characterized in our task but not in the revenue estimation task. Pronounceable company names induce different expectations about company stock performance than non-pronounceable names [49], and these expectations could be reflected in a variety of likelihoods that cause the resulting estimates to resemble those coming from a mixture of decision functions. This is of course speculative, and the divergence may be due to other task differences, but it highlights the importance of characterizing all of the components of Bayesian decision theory.

Overall, our findings for the decision function roughly correspond with those found in continuous estimation, so there seems to be no strong dividing line between the decision function used to make these two types of estimates. Of course decision functions have usually not been characterized in much detail nor have been characterized across a range of tasks, so later investigations could reveal subtle differences in how the task shapes the decision function used.

In terms of the prior, across three experiments we found the novel result that participants were better characterized as using a categorical prior than by a simple parametric distribution or by a kernel density estimate with any appreciable width. Mixture priors could possibly explain the results of Experiments 1 and 2, but Experiment 3 showed that a simple implementation of a mixture prior did not match the data as well for most participants.

The use of a categorical prior was supported by participants’ ability to learn complex multimodal distributions very quickly. The speed and flexibility of participants’ prior learning stands in contrast to work in continuous tasks, where it is difficult to find evidence for quick learning of bimodal priors. One task required 4,000 feedback training trials to teach participants a bimodal distribution [10], and another required 1,700 trials [22]. However, when [19] used 1,500 training trials in an interval timing task, there was some suggestion of bimodality if the peaks were well-separated, but the data could also be explained by a uniform prior.

The only example of equally fast learning of a bimodal prior without giving participants hints comes from other experiments using discrete numerical responses. In the revenue estimation experiments of [18], it was found that a bimodal prior distribution could be reconstructed within 400 training trials. More impressive was the demonstration that bimodal priors could be reconstructed after as little as 12 trials by individual participants [46]. However, these demonstrations come from tasks in which participants are asked to reconstruct the distribution rather than make an estimate, so this work is the first to show that participants do use bimodal priors when making estimates, and can learn to do so more quickly than in continuous tasks. In addition the priors that participants learned were impressively accurate: we showed that quadrimodal prior distributions could be learned, and that their priors were better described by a categorical distribution rather than a kernel density estimate or some forms of mixture models.

Given these differences in speed of learning, it is interesting to speculate whether there are particular properties of tasks that require discrete numerical responses that make it easier to learn a complex prior. One difference between discrete numerical and continuous estimates is that it is easier to provide clear feedback for discrete numerical estimates. Both [19] and [10] used visual position as feedback in their sensorimotor and interval timing tasks, and noise in vision and memory makes this feedback less certain. In the orientation estimation task of [22] participants were told their average deviation every 20 trials, while this feedback is digital it does not provide as much information as the true orientation used on each trial. It is difficult to see how the feedback could be improved for tasks that require continuous responses: feedback either needs to be susceptible to noise (perhaps both sensory noise and noise in encoding and remembering the feedback) or it is not directly mapped to the responses. Participants cannot perfectly be shown what response they should have made.

In contrast, our experiments and the experiments of [18] showed participants the correct response after every trial in essentially a noise-free fashion. This is a real advantage of using discrete numerical responses and feedback because feedback can be given uncorrupted by sensory noise after every trial and it is easily mapped to the responses than participants make. In fact, [46] explicitly showed this difference when participants were asked to reproduce a distribution: for experiments in which numbered stimuli were replaced by circles of various sizes, participants required more trials and greater separation between the modes to learn the bimodal distributions. It is possible that the differences in clarity of feedback explain the rates at which participants learn bimodal priors in different tasks. If participants are using a form of Occam’s razor when constructing their prior distribution, then the more informative trials would more quickly convince them to abandon a simpler prior in favor of a more complex representation.

### Implications for learning of priors

The priors learned in our experiments, especially Experiment 3, were much more complex than those taught to participants in other estimation tasks [10, 15, 18, 19, 22, 46]. In addition to having four modes, our prior had a pattern of low-probability and no-probability responses that participants’ responses matched. Participants were not just representing the prior as a mixture of two parametric components, but were learning the prior probabilities associated with individual responses.

Work using other tasks has demonstrated fairly complex prior learning, but in other tasks it is generally not clear whether participants are learning a prior or a mapping. For example using a categorization task, a subset of participants learned to discriminate a multidimensional quadrimodal distribution from a multidimensional mixture of two Gaussian distributions [50]. While participants were able learn these complex discriminations and their behaviour could be described by a model that approximates Bayesian inference, this work did not rule out a complex decision bound model (i.e., a mapping) as an alternative [50, 51].

In Experiment 3 we ran additional trials to test whether participants were actually learning and using a prior or if instead they were learning a mapping from the stimuli to the responses. As discussed in the S1 Methods, we found that the responses of more than half of participants were best explained by a prior rather than a mapping. Use of a prior is also supported by recent work that demonstrated that participants take into account the reliability of various senses in a multisensory numerosity task [38].

Our results contrast with other work showing that participants do not learn a categorical prior. In a continuous estimation task with a wide range of possible responses, a categorical prior did not explain the data as well as a mixture [15]. Likewise in a numerosity task that showed participants a much wider range of numbers than our experiments, a mixture model provided a better fit to their participants’ data than just using the trained examples as a prior [52].

The key difference is likely the variety of correct responses in each experiment. As the number of potential responses increases it is hard to imagine that participants would precisely track the frequency with which every single number appeared. For example, if every number from 100 to 200 appeared in a random order with the exception of number 134, it is implausible that participants would notice.

This contrast raises questions about where the transition between a categorical prior and a mixture model occurs, and even if there is a distinction between the two. It is possible that participants represent a small set of numbers symbolically and use a categorical prior, but represent a large set of numbers as a mixture prior over a continuous variable. Alternatively, it could be that our categorical prior is simply a mixture with a separate component for each response. In this case, there would likely be a smoother transition between representations of the prior for small and large sets of responses.

### Implications for how people perform inference

Very few of our participants were best fit by a simple decision function: the max or mean of the untransformed posterior distribution. Instead it appeared that the large majority of participants were performing some kind of approximate inference by drawing one or more samples. Previous work has put forward mechanisms with which this could be done. For example, [20] showed that participants’ responses were consistent with either taking the mean of a number of samples in a continuous estimation task or drawing a single sample from an exponentiated posterior distribution. Later, [15] disambiguated these two operations with a bimodal prior, showing that raising the posterior distribution to an exponent was the better description.

Both of these mechanisms have been touted as tradeoffs between effort and accuracy, and possibly a rational use of cognitive resources [35], though there is always the possibility that participants have particularly complex hypotheses about the computer’s behavior instead. Drawing a sample may take time or effort, and a small number of samples may provide the best tradeoff between effort and accuracy to yield the highest overall reward [36]. Similarly, raising a posterior distribution to a power has also been cast as a tradeoff between effort and accuracy, but one that assumes effort is required to perform the exponentiation that transforms the belief distribution into a response distribution [53].

While this makes for a nice contrast, the picture is complicated by two additional mechanisms that are essentially indistinguishable from exponentiating the posterior distribution, even for bimodal priors: taking the maximum of a number of samples drawn from the posterior distribution, and taking the maximum of a posterior distribution that has been corrupted with noise [15]. This last mechanism may well differ from the others if it is assumed that the amount of noise in the posterior is not under the control of the participant; in this case sampling-like behavior would not be a tradeoff between effort and accuracy.

We add to this literature by showing that while an exponentiated posterior distribution is necessary to explain the data as in [15], additionally a large number of participants appear to be taking the mean of a number of samples drawn from this exponentiated posterior distribution. Despite the fact that the maximum of the posterior was asked for by identifying only exactly correct responses as ‘correct’, participants still showed some tendency to produce some responses near the mean of the posterior.

It is interesting to speculate what sort of mechanism could support both a tendency to respond with the max and a tendency to respond with the mean. Our best fitting combination of the mean of samples from an exponentiated posterior distribution is one possibility. It could be that participants are using an exponentiated posterior helps to emphasize the mode which is most likely under the posterior distribution. The later sampling operation helps to select the best response in that mode, trading off the need to pick the highest posterior with the uncertainty introduced by having several highly likely responses in close proximity. It may even be that responses near the mean of the posterior are an accidental byproduct of this two-stage process.

However, the difficult-to-distinguish alternatives to an exponentiated posterior point toward alternative combinations. One of these is a pure sampling approach: participants draw samples from the posterior distribution and sometimes take the maximum of the samples and sometimes take the mean. Another alternative combination is to ascribe all the variability to noise in the posterior distribution: using a noisy posterior distribution, sometimes participants take the maximum and sometimes they take the mean.

To gain additional purchase on this question, we correlated the average response times of participants with the model parameters. It might be expected that if participants were using any of tradeoffs between effort and accuracy that there would be correlations between each participant’s average response time and the number of samples or the exponent that the super-model recovered. This kind of correlation has been found in previous work when looking two-alternative responses [36]. However, both within each experiment and across experiments, we found no reliable relationship between the either of these model parameters and the response times of participants (see S1 Methods for details).

On the surface, this null result could be considered evidence that participants use the maximum or mean of a noisy posterior distribution to produce their estimates and that the amount of noise in the posterior does not depend on participant effort. However, it could also be that participants have such differing goals for effort / accuracy tradeoffs that this washes out whatever correlations there are between response time and model parameters. Future work would provide stronger tests of these mechanisms using within-participant designs that manipulate rewards and time-pressure, along with emphasizing that the computer is not responding to participant behavior.

### Connections to everyday tasks

There are many examples of discrete numerical tasks in everyday life, such as the examples of painters quickly assessing the size of a wall in order to buy the right number of paint cans or of the party hosts assessing the hunger of their guests when buying a discrete number of pizzas. In our experiments, we used a numerosity task and an area estimation tasks because both of these tasks are well studied and the likelihood distributions have been well characterized. This allowed us to quickly measure the standard deviation of the likelihood for each participant. If we had used less controlled stimuli, then we might have had to measure the full distribution of responses that each individual stimulus evoked in order to characterize the likelihood.

Our laboratory tasks are similar to some everyday tasks. The numerosity task we used is similar to estimating the number of visible stars in the sky (which does vary depending on the time of day and light pollution), and estimating the size of a rectangle shares some similarities to the example of the painter who needs to assess the area of wall. However, there are differences as well: the stars in the sky do not differ in size from night to night and painters can view a wall from many distances and angles before producing an estimate. With the right stimuli, it would be interesting to investigate real-life performance in discrete numerical estimation tasks.

### Conclusions

Our results demonstrate that people represent a surprising amount of complexity in their prior distribution with relatively few training trials and use this complex prior when making new estimates. Training complex priors has multiple benefits: we can more easily observe how people represent priors and we can investigate some of the more complex schemes describing how people convert the posterior distribution into a single estimate.

This work raises many questions about how prior and posterior distributions are represented and how estimates are made. Discrete numerical estimation tasks, which are simple to implement and quick to train, are well suited for future work in this area.

## Methods

### Experiment 1

Twenty-one University of Warwick students participated in this experiment for course credit. Participants gave written informed consent and the experiment was approved by the University of Warwick Humanities and Social Sciences Research Ethics Committee. Participants were divided into three groups and each participated in one version of the experiment, as outlined below. One participant was excluded because of computer error and second was only given one block of discrimination trials but was included in the analysis.

The stimuli consisted of displays of a number of identical dots. Each display of dots consisted of white dots on a black background, visible for 500 ms. Dot radius and dot density were randomized for each display to encourage participants to make numerosity judgments instead of judging the amount of light produced by the display, the density of the display, or the area occupied by the dots. In a single display all dots had a single common radius of between 3 and 9 pixels that was chosen randomly with equal probability on each trial. Dots were positioned randomly within a circular available region which was centered on the display, subject to the constraint that no dot could lay within one-dot-diameter of another dot. The available region randomly varied between 150 and 450 pixels in radius. A uniform draw was made over the possible values of dot density (where density equaled the maximum number of dots that could appear in that block divided by the area of the available region), which determined the radius of the available region on a trial.

The experiment consisted of a single session with three blocks. The estimation trials, in which participants saw a single display of dots and responded with their estimate of the number of dots in each display, were presented in the second block. The estimation trials differed for the three groups of participants: the narrow group, the medium group, and the wide group. The narrow group consisted of eight participants who saw 800 estimation trials in which the number of dots varied between 23 and 29. For this group, displays with 23 and 29 dots each appeared with probability 0.3 and the displays with the remaining numbers appeared with probability 0.08. The medium group consisted of six participants who saw 700 estimation trials in which the number of dots varied between 23 and 32. For this group, displays with 23 and 32 dots each appeared with probability 0.3 and the displays with the remaining numbers appeared with probability 0.05. The wide group consisted of six participants who saw 700 estimation trials in which the number of dots varied between 23 and 35. For this group, displays with 23 and 35 dots each appeared with probability 0.28 and the displays with the remaining numbers appeared with probability 0.04. Every participant saw 10 practice estimation trials displaying between one and four dots before beginning the main phase of the experiment.

The first and third block consisted of 128 discrimination trials each (always proceeded by 4 practice trials), in which participants saw two sequential displays of dots and picked the display that contained the larger number of dots. On every discrimination trial, one of the displays had a specific high or low number of dots. These anchor numbers were set to be either 11 dots below or above the lowest number seen in the estimation trials. The other display consisted of a number of dots that was equal to the anchor plus an offset. The offset was randomly chosen with equal probability from the set of {-8, -4, -2, -1, 1, 2, 4, 8}. Because of computer error one participant, in the group that had a range of 23 to 29 in the estimation trials, was given anchor trials of 18 and 54, in his or her first block. This participant received the correct anchor trials (12 and 40) in the third block.

On estimation trials, after the dot display disappeared, participants were asked to enter the number of dots that they saw. After entering their response, participants received feedback about whether they were correct and the actual number of dots that were shown. On discrimination trials, participants only received feedback about whether they were correct or not.

### Experiment 2

Twelve University of Warwick students participated in this experiment for £6 apiece. Participants gave written informed consent and the experiment was approved by the University of Warwick Humanities and Social Sciences Research Ethics Committee. Participants were divided into two groups and each participated in one version of the experiment, as outlined below.

The stimuli consisted of displays of rectangles of particular areas. For each display, the width of the rectangle and its position were randomized to encourage participants to judge the area of the rectangles without exclusively relying on its length, width, or position on the screen. Rectangle width was chosen from a continuous uniform distribution between 2cm and 10cm, with length chosen to achieve the desired area given the width. A fixed positional jitter was chosen for each trial uniformly from a 6cm square. On estimation trials the rectangle was on average in the center of the screen and appeared for 500ms. On discrimination trials, the first rectangle appeared 10cm left of center plus positional jitter for 500ms, and after a 500ms delay the second rectangle appeared 10cm right of center plus positional jitter for 500ms.

The procedure was identical to Experiment 1 with the following exceptions. Participants responded in the estimation trials with the area of the rectangle in square cm. A narrow group and a medium group was run in this experiment consisting of six participants each, with equivalent numbers and probabilities to the groups with the same names in Experiment 1. Every participant saw 700 estimation trials in this experiment.

### Experiment 3

Twenty-four University of Warwick students participated in this experiment for £6 apiece. Participants gave written informed consent and the experiment was approved by the University of Warwick Humanities and Social Sciences Research Ethics Committee. Participants were divided into three groups and each participated in one version of the experiment, as outlined below. Two participants were excluded from the rectangle group and one from the easier numerosity group because of computer error. Two additional participants from the easier numerosity group saw between ten and fifteen additional non-feedback trials at the beginning of the second estimation block and their data (excluding these trials) were included in the analyses.

For all groups the experiment consisted of four blocks: the first discrimination block of 128 trials, the first estimation block of 500 trials, the second estimation block of 200 trials, and finally the second discrimination block of 128 trials. Feedback was given for all blocks except for the second estimation block which served as a test of whether a prior had been learned. The first discrimination and estimation blocks used easier-to-see displays than the second discrimination and estimation blocks. The three groups of participants differed in the details of the displays they were shown. The difficult numerosity group were run with the same display parameters as in Experiment 1 during the first discrimination and estimation blocks. During the second estimation and discrimination blocks, the first numerosity group was shown each dots display for 50ms and at a much reduced luminance. The easier numerosity group was given different display parameters during the first discrimination and estimation blocks: the range of the common radius of dots was from 6 to 9 pixels, while the available region randomly varied between 225 and 375 pixels. The easier numerosity group had a shorter display (50ms) as well as more variability on the second discrimination and estimation blocks: the range of the common radius of dots was from 1 to 11 pixels, while the available region randomly varied between 150 and 450 pixels. The third group, the rectangle group, was given rectangles that randomly varied in width between 4.8 and 6.5cm during the first discrimination and estimation blocks. In the second discrimination and estimation blocks, this group was given a shorter (50ms) and slightly dimmer (gray instead of white) display with rectangles that randomly varied in width between 2 and 10cm.

All participants in this experiment were given the same trial structure during the estimation trials. The distribution that generated the trials was quadrimodal with a 20% chance of drawing each of the numbers 23, 25, 29, or 31. In addition, there was a 10% chance of drawing each of the numbers 24 and 30.

### Analysis—Discrimination data

To estimate the variability in participants’ internal estimates, *X*, we analyzed the discrimination data in order to utilize fitted parameters for the estimation task. Specifically we assumed that the internal estimate was distributed according to a log-normal distribution (in accordance with Weber’s law) log(*X*)∼*N*(log(*X*); log(*S*), *σ*^2^) where *X* and *S* are positive integers. Participants were presented with two stimuli, *S*_1_ and *S*_2_ (as in the 2AFC discrimination trials) and had to estimate which one was larger.

In order to fit the variable *σ* we maximized the log-likelihood across trials (or rather minimized the negative log-likelihood) using Matlab’s fminbnd function $\hat{\sigma }=arg max\Sigma_{i}\log P\left(R_{i}|S_{i,1:2},\sigma \right)$. The model likelihood *P*(*R*_*i*_|*S*_*i*,1: 2_, *σ*) was estimated numerically for each trial and condition by sampling *X*_1_ and *X*_2_ 10,000 times and for each set generating a fictitious response. *P*(*R*_*i*_|*S*_*i*,1: 2_, *σ*) = (1/10000)Σ_*l*_ *H*(*X*_*l*,1_ − *X*_*l*,2_) where H is the Heaviside function and log(*X*_*l*,1_)∼*N*(log(*X*_*l*,1_); log(*S*_1_), *σ*^2^) and log(*X*_*l*,2_)∼*N*(log(*X*_*l*,2_); log(*S*_2_), *σ*^2^) are samples from the generative model above. This analysis assumed that participants chose the most likely response on each trial, which is what was found in a recent analysis of 2AFC choice tasks [54].

### Analysis—Estimation data

The purpose of our analysis for the Estimation data is to compare and rule out different models of human decision making (see Experiments 1–3 above). One common way of comparing perceptual models of different number of parameters (see e.g. [55]) is to fit the parameters of each model through maximum likelihood and compensate for differences in model complexity by calculating the Bayesian Information Criterion (which penalizes models with large number of parameters).

Our secondary analysis instead created a single model that encompasses all of the candidate models as special cases, that is for certain parameter sets the larger model is equivalent to each of the nested candidate models. The best fit of the parameters therefore shows which of the models best describes the data.

We will first describe the generative model, how to perform inference over it, how to perform model comparison, and lastly we describe the ‘super-model’ and explain what specific models it encompasses.

#### Generative model

Participants on trial *t* are presented with *S*_*t*_ number of dots drawn from a fixed categorical distribution *P*(*S*_*t*_) (see e.g. Fig 1). In accordance with our description above participants have an internal estimate *X*_*t*_ given by log(*X*_*t*_)∼*N*(log(*X*_*t*_); log(*S*_*t*_), *σ*^2^). This naturally leads to larger absolute variability in *X* for larger *S*.

We assume that our participants have an understanding (at an intuitive level) of the process and are using Bayes’ rule to infer the best estimate of the number of dots $\hat{S_{t}}$ given their chosen decision function.

#### Inference

Optimal inference requires combining the likelihood and prior expectations about *S*_*t*_ using Bayes’ rule: *P*(*S*_*t*_|*X*_*t*_) ∝ *P*(*X*_*t*_|*S*_*t*_)*P*(*S*_*t*_).

Based on the generative model the likelihood of *S*_*t*_ is given by

$$P\left(X_{t}|S_{t}\right)=N\left(\log\left(S_{t}\right);\log\left(X_{t}\right),\sigma^{2}\right)\propto \exp\left(\frac{-{\left(\log\left(X_{t}\right)-\log\left(S_{t}\right)\right)}^{2}}{2\sigma^{2}}\right)$$ (3)

where *S*_*t*_ is restricted to be integer values. This same representation was used in both the numerosity and rectangle area tasks.

The prior is *P*(*S*_*t*_) = ∫*P*(*S*_*t*_|*ϕ*)*P*(*ϕ*)*dϕ* where *P*(*S*_*t*_|*ϕ*) is a discrete categorical distribution and *P*(*ϕ*) is a Dirichlet distribution:

$$P_{\alpha }\left(\phi \right)=f\left(\phi_{1},\cdots ,\phi_{K-1};\alpha_{1},\cdots ,\alpha_{K}\right)=\frac{1}{B(\alpha )}\prod_{i=1}^{K}\phi_{i}^{\alpha_{i}-1}$$ (4)

where *α*_*i*_ are the parameters of the distribution and *B*(*α*) is the multinomial Beta function. The prior is then $P\left(S_{t}=i\right)=\frac{\alpha_{i}}{\sum_{j}\alpha_{j}}$ which is a discrete categorical distribution (sometimes referred to as a multinomial with just one draw).

A property of P(ϕ) is that it is conjugate with the categorical distribution. This means that if categorical data *S*_*true*_ = **K** is observed (in terms of feedback provided to participants) the prior over *α* is updated as:

$$P_{\alpha^{\prime }}\left(\phi \right)\propto P_{\alpha }\left(\phi \right)P_{K}\left(S_{true}\right)$$ (5)

where *P*_*K*_(*S*_*true*_) is a categorical distribution with 1 at the observed value and 0 elsewhere. The new set of parameters are **α**^**′**^ = **α** + **K**.

Hence updating the prior after trial *t* merely requires increasing the variable *α*_*t*, *i*_ = *α*_*t* − 1, *i*_ + 1 corresponding to the feedback value *i*. We will refer to this as the *Dirichlet updating prior*.

However it is possible that this updating is not done efficiently, leading to a broader updating that encompasses neighboring values. To allow for this we update according to $\delta \alpha_{j}=\frac{N(j;i,\psi^{2})}{\Sigma N(j;i,\psi^{2}))}$ (where *i* and *j* are integers). We will refer to this as a *kernel density update prior*. For *ψ* less than 0.0115 about 95 percent of the mass of the kernel would be placed at the location of feedback *i*, $\int_{i-1}^{i+1}N\left(\log\left(j\right);\log\left(i\right),{\left(0.0115\right)}^{2}\right)dj\approx 0.95$ (for the largest value *j* = 32 presented in the experiment), thus we regain the previously described Dirichlet prior update mechanism as a special case. In contrast a very large *ψ* would indiscriminately update all values.

Finally, to transform the posterior into a response, three typical decision functions were compared for our Model Comparison (see below): *Max* ($\hat{S_{t}}={arg max}_{S}P_{n}\left(S_{t}|X_{t}\right)$), *Mean* ($\hat{S_{t}}=\sum_{i}mP_{n}\left(S_{t}=m|X_{t}\right)$) or *Sampling* ($\hat{S_{t}}=s^{\prime }$ where *s*′ is a sample from the transformed posterior: *s*′ ∼ *P*_*n*_(*S*_*t*_|*X*_*t*_)).

### Model comparison

A traditional way of comparing models is by maximizing the likelihood of each model and correcting for number of parameters using the Bayesian Information Criterion [48]. We factorially combined two different priors (Dirichlet or Gaussian kernel), three types of decision function (mean, max or sampling) and three types of noise models (none, trembling hand or softmax) to produce 16 different models for each participant (note that softmax is not defined for a max decision function removing two of the 2x3x3 = 18 combinations).

The priors were updated using either a variable width Gaussian kernel or “zero width” Dirichlet updating. Combined with the likelihood, this creates the posterior distribution *P*(*S*_*t*_|*X*_*t*_) upon which the subject bases their decision.

A softmax noise model performs a transformation of the posterior

$$P_{n}\left(S_{t}=i|X_{t}\right)=\frac{P{\left(S_{t}=i|X_{t}\right)}^{\beta }}{\Sigma_{j}P{\left(S_{t}=j|X_{t}\right)}^{\beta }}$$ (6)

where *β* < 1 leads to a widening (or flattening) of the posterior, while *β* > 1 leads to a sharpening of the posterior. For noise models none or trembling hand we set *β* = 1.

Choices (as discussed above) are then made based on either max, mean (average), or sampling of the exponentiated posterior. Finally, the trembling hand noise model, included as an alternative to the softmax model, states that participants have a small probability *ϵ*, of performing a random choice. I.e. $\hat{S_{t}}∼U\left[1:100\right]$ if *e* < *ϵ*, where *e* is randomly sampled from *U*[0: 1]. While the trembling hand noise model is structurally implemented after the decision is made, it has a similar effects as the softmax noise model in increasing the variability of the responses.

As the internal variable *X*_*t*_, was unknown to the experimenters we used ancestral sampling [28], drawing 10,000 samples from the generative process for *X*_*t*_ ∼ *P*(*X*_*t*_|*S*_*t*_) followed by the inference by the subject as described above (based on any model parameters). This generates 10,000 independent estimates of $\hat{S}$ which we can use to numerically approximate the probability of response $\hat{S_{t}}$. As they are essentially discrete counts we describe them as a discrete categorical distribution which provides us with the model likelihood $P_{m}\left(\hat{S_{t}}|S_{t},par\right)$ for trial *t* and any model *m* and its associated parameters *par*.

For each model and parameter set for each subject the log-likelihood was thus calculated as $\log L=\Sigma_{t}\log P\left(\hat{S_{t}}=R_{t}|S_{t},par\right)$. Note that the parameter *σ* had been fit independently for each subject through the discrimination experiment above and was thus not a free parameter. Any trials with subject responses of less than 5 dots (*R*_*t*_ < 5) were ignored as erroneous key presses given that the true number of dots presented were at least 23. Furthermore to avoid any singularities in calculating the likelihood we allowed for a small probability (0.001) that participants would make a random response in the range [1:100] (similar to a slight Trembling Hand).

Any parameters *par* = (*β*, *ϵ*, or *ψ*) were fit through maximum likelihood (Matlab’s fminsearch). For model comparison we calculated BIC for each subject and each model:

$$BIC=-2*\Sigma_{t}\log P\left(\hat{S_{t}}=R_{t}|S_{t},par\right)+N*\log\left(M\right)$$ (7)

where N is the number of model parameters in *par* (0, 1 or 2) and *M* is the total number of trials.

### Parameter fitting (‘Super-model’)

As an alternative to the model comparison we can compare models factorially based on how they update the prior (given by parameter *ψ*), how many samples are drawn (parameter *n*), and how exponentiated the posterior is (parameter *β*). The fitted parameter set for each subject encapsulates which model aspects best explain the subject’s behavior. Thus the specific models compared above become special cases within this larger parameter space, allowing us to extrapolate between the models.

We put all of these into a unified framework, which we refer as the ‘Super-model’ (as other models are sub-sets of it). Given the posterior *P*(*S*_*t*_|*X*_*t*_) we assume that participants choose their response by averaging over samples:

$$\hat{S_{t}}=\frac{1}{n}\Sigma_{i}^{n}s_{k}$$ (8)

where the samples *s*_*k*_ are given by

$$s_{k}∼P_{n}\left(S_{t}=i|X_{t}\right)=\frac{P{\left(S_{t}=i|X_{t}\right)}^{\beta }}{\Sigma_{j}P{\left(S_{t}=j|X_{t}\right)}^{\beta }}$$ (9)

where the parameters *n* and *β* are fitted for each subject (see below).

The summation over samples allows us to approximate properties of specific decision functions. For *n* = 1 a single sample is drawn, equivalent to the *sampling decision function*. The averaging approximates the mean of the distribution for very large *n* (thus approximating the *mean decision function*).

The sampling of *s*_*k*_ is from a softmax function (also known as the exponentiated Luce choice rule [56, 57]) which causes all the probability density to be sharpened at the peaks of the posterior for larger values of *β*. For large values of *β* the number of samples (*n*) becomes of little consequence (for example for *β* > 2.7, with *X*_*t*_ = 23 and *σ* = 0.22 after learning for 300 trials, more than 95 percent of the probability is at the maximum a posteriori).

In this way specific parameters emulate the mean (average, *n* = 10000), max (*β* = 1000) and sampling (*n* = 1) decision functions.

In order to fit the variables (*ψ*, *n*, *β*) we performed log-likelihood maximization on *ψ*, *β* using Matlab’s fminsearch function (on −log*L* with 5 random initializations), for each of *n* = [1, 2, 3, 4, 5, 10, 30, 100, 1000]. For each subject this allowed us to find the parameter set with the maximum likelihood and, given that the models of interest are nested models of this model-parameter set, indirectly find the model that best describes the data.

## Supporting Information

S1 Methods Includes an investigation of whether a prior or mapping is used, the individual results of model fits, a model verification exercise, and correlations between response time and model parameters. (PDF)

S1 Dataset The discrimination and estimation data from every reported participant in all experiments in Matlab format. (ZIP)
