# Supplementary material for: Fast and Accurate Learning When Making Discrete Numerical Estimates
Source: PLoS Comput Biol. 2016 Apr 12;12(4):e1004859. doi: 10.1371/journal.pcbi.1004859 (PMC4829178; doi:10.1371/journal.pcbi.1004859)
Supplement: S1 Methods — (PDF) [file pcbi.1004859.s001.pdf]

Supplemental Methods for "Fast and accurate learning when making discrete numerical estimates"

Adam N. Sanborn[1], Ulrik R. Beierholm[2]
1 Department of Psychology, University of Warwick, Coventry, UK
2 Centre for Computational Neuroscience and Cognitive Robotics, University of Birmingham, Birmingham, UK
∗ E-mail: Corresponding a.n.sanborn@warwick.ac.uk

Individual participant super-model parameters and model fits

This section gives the individual parameters fit to each of the participants across the three experiments. Table 1 gives the parameters for the participants in Experiment 1, Table 2 gives the parameters for the participants in Experiment 2, and Table 3 gives the parameters for the participants in Experiment 3.

In each of the tables, group names correspond to the group names used in the main text and participant numbers correspond to the participant numbers attached to the raw anonymized data. Parameters σ_1 and σ_2 represent the standard deviation parameters of the lognormal distributions from the first and second discrimination sessions respectively. For fitting purposes in Experiments 1 and 2, the mean of σ_1 and σ_2 was used, but for Experiment 3, because the discrimination sessions were different, σ_1 was used for the fitting.

For the super-model, n is the best-fitting number of samples taken from a posterior distribution exponentiated by β which itself is updated by a Gaussian kernel with standard deviation ψ. For large values of β the number of samples (n) becomes of little consequence (for example for $\beta > 2.7$, with $X_t = 23$ and $\sigma = 0.22$ after learning for 300 trials, more than 95 percent of the probability is at the maximum a posteriori). For ψ less than 0.0115 about 95 percent of the mass of the kernel would be placed at the location of feedback i, $\int_{i-1}^{i+1} N(\log(j); \log(i), (0.0115)^2) dj \approx 0.95$ (for the largest value $j = 32$ presented in the experiment), thus we have the Dirichlet prior update mechanism as a special case.

For the model comparison, the three-letter model code indicates sequentially the type of prior (Dirichlet or Gaussian kernel), the decision function (Average, Max, or Sample), and the noise model (None, Softmax, or Trembling hand). A comparison of model posterior probabilities that first converts BIC values into Bayes factors [1] and then assumes equal priors for each of the models is shown in Figure 1 for the None models only and in Figure 2 for the full set of models. The parameter ψ was the standard deviation of the Gaussian kernel (if applicable) and β was the exponent applied to the posterior distribution before applying the decision function. Model details are given in the Materials and Methods section of the main text.

Model verification

A crucial part of any type of computational modeling is some aspect of verification that the model is in fact performing its true role. This is not just to guard against errors in the programming of the model, but also to avoid problems in the specification of the model itself [2]. A standard way to do this is by feeding the model data that has been generated through a known process and known parameters. In our case we chose 5 parameter sets (see table 4) to characterize 5 fictional subjects, and simulated subject responses to 3 sets of stimuli. For each of these fictional data sets we could thus perform inference based on the hierarchical 'super-model', to infer the most likely set of parameters to have generated the data set, which were then compared to the parameters that generated the data.

Most of the inferences on the data sets recovered parameter values close to the original, except for set 14 where the inference process lead to erroneous values of n and β (although note that for large values of softmax β the number of samples from the posterior n becomes irrelevant).

Table 1. Discrimination fits, super-model parameters, and best-fitting model for Experiment 1 (numerosity estimates)

		Discrimination		Super-model			Best-fitting model		
Group	Participant	σ_1	σ_2	n	ψ	β	Model	ψ	β
Narrow	2	0.255764	0.204191	2	0.0001	2.6961	DSS		4.5477
Narrow	3	0.295348	0.134957	2	0	1.8313	DAS		1.7626
Narrow	4	0.218559	0.174174	4	0.0031	2.1886	DAS		2.8893
Narrow	5	0.230179	0.333471	4	0.0283	4.7552	DAS		2.6385
Narrow	6	0.446284	0.175661	30	0.0062	4.5669	DAS		5.1194
Narrow	7	0.27596	0.170674	30	0.0075	3.3752	DAS		4.6332
Narrow	8	0.889141	0.502028	3	0.0026	1.9411	GAS	0.0167	3.5715
Narrow	9	0.347254	0.16768	3	0.0042	2.3213	GAS	0.0165	6.2195
Wide	10	0.143747	0.168133	1	0.0013	1.4019	DSS		3.5988
Wide	11	0.535208	0.213504	1	0.0016	1.6442	DSS		1.4019
Wide	12	0.258696	0.189626	1	0	1.549	DSS		1.6442
Wide	13	0.447938	0.222017	30	0.0036	3.8028	DAS		1.5685
Wide	14	0.22741	0.254361	30	0.0067	4.1066	DAS		4.0837
Wide	15	0.206573		1	0.0058	1.3183	DSS		4.5967
Medium	16	0.146783	0.094184	1	0.0006	1.0384	DSN		
Medium	17	0.307802	0.466872	1	0.0029	1.956	DSS		1.3503
Medium	18	0.236975	0.594877	1	0.002	1.8375	DSS		1.956
Medium	19	0.280635	0.147977	1	0	3.5404	DSS		1.8375
Medium	20	0.213696	0.132815	1	0.0027	1.1838	DSS		3.39
Medium	21	0.153447	0.177586	2	0	1.8159	DSS		1.1837

Table 2. Discrimination fits, super-model parameters, and best-fitting model for Experiment 2 (rectangle estimates)

		Discrimination		Super-model			Best-fitting model		
Group	Participant	σ_1	σ_2	n	ψ	β	Model	ψ	β
Narrow	1	0.256892	0.677599	3	0.0062	3.2784	DSS		1.3607
Narrow	2	0.44897	1.984764	2	0.006	2.3872	DSS		1.4944
Narrow	3	0.407995	0.261767	30	0.0021	8.9238	DAS		8.8158
Narrow	4	0.45104	0.701805	30	0.0032	7.5965	DAS		7.8321
Narrow	5	0.408036	0.419497	100	0.0058	5.7903	DAS		6.1005
Narrow	6	0.373962	0.186244	3	0.0045	2.8674	DSS		1.4711
Medium	7	0.210494	0.426135	4	0.0376	7.4426	DSS		1.7821
Medium	8	0.195323	0.284592	4	0.0091	2.9075	DSS		1.5451
Medium	9	0.744606	0.963194	10	0.0073	6.4477	DAS		7.2107
Medium	10	0.491546	0.305074	100	0.0014	10.6737	DAS		10.7427
Medium	11	0.410943	0.204927	3	0.0039	2.215	DAS		3.5391
Medium	12	0.286218	0.331675	5	0.005	3.4895	DSS		1.5939

Table 3. Discrimination fits, super-model parameters, and best-fitting model for Experiment 3

		Discrimination		Super-model			Best-fitting model		
Group	Participant	σ_1	σ_2	n	ψ	β	Model	ψ	β
Difficult Numerosity	30	0.661596	0.80638	1	0	1.8588	DSS		1.8588
Difficult Numerosity	31	0.133094	310.9089	1	0.0111	0.9984	DSN		
Difficult Numerosity	33	0.18643	263.4332	1	0.0212	1.0029	GSN	0.0212	
Difficult Numerosity	34	0.172183	1.43462	1	0	2.3892	DSS		2.3362
Difficult Numerosity	35	0.196892	0.327686	1	0.0032	1.5643	DSS		1.5784
Difficult Numerosity	37	0.220094	6.684557	1	0.0056	2.7584	DSS		3.574
Easier Numerosity	42	0.141649	0.237402	1	0.0031	1.076	DSN		
Easier Numerosity	43	0.324717	0.327129	1	0.147	5.4118	GMN	0.1842	
Easier Numerosity	44	0.119111	0.253625	1	0.0038	1.3383	DSS		1.2409
Easier Numerosity	45	0.174715	0.174942	3	0.0015	1.503	GMN	0.0769	
Easier Numerosity	46	0.128786	0.595294	1	0	1.3191	DSS		1.3191
Easier Numerosity	47	0.30413	0.24329	1	0.0015	1.4895	DSS		1.4895
Easier Numerosity	48	0.110992	0.186132	1	0.0019	1.7776	DSS		1.7776
Rectangle	42	0.146812	0.196617	1	0.0019	1.6655	DSS		1.6684
Rectangle	43	0.108555	0.204477	1	0.0031	2.0154	DSS		2.0154
Rectangle	45	0.140943	0.195616	4	0.0026	2.046	GAS	0.0523	2.5816
Rectangle	46	0.161395	0.259085	30	0.0257	23.3048	DSS		1.4086
Rectangle	47	0.19182	0.23457	1	0.0128	1.0025	DSN		
Rectangle	48	0.111025	0.161686	1	0.0148	0.997	GSN	0.0149	
Rectangle	49	0.13177	0.113415	1	0.0004	0.9608	DMT		0.0349
Rectangle	50	0.270577	0.512169	1	0	1.7699	DSS		1.6709

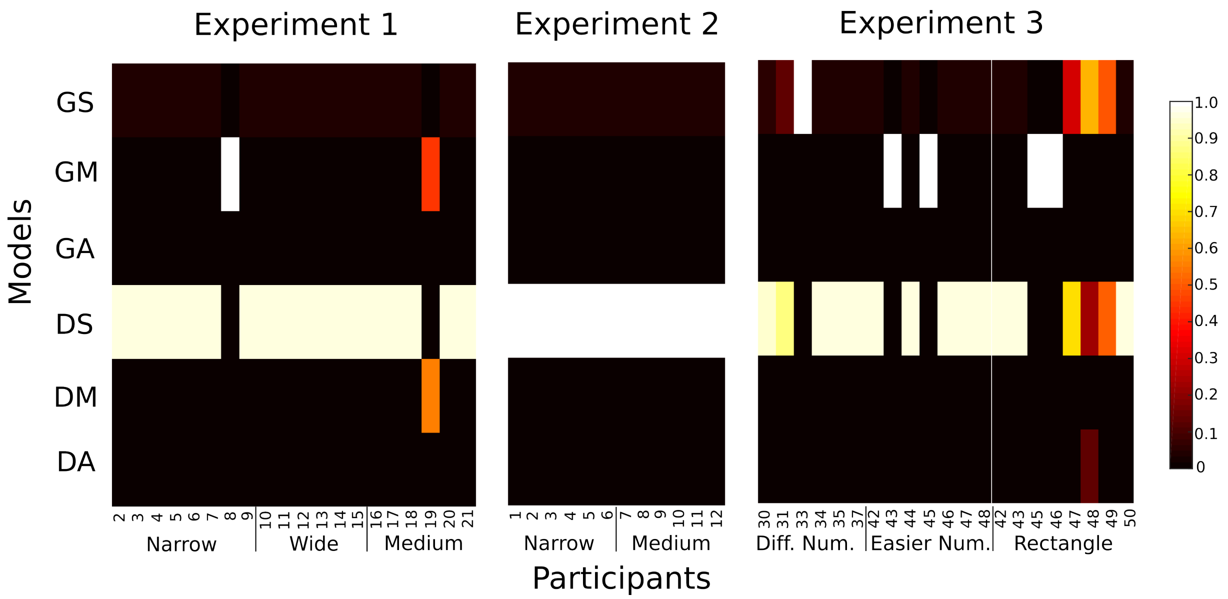

Figure 1. Heat map of the model posterior probabilities based on BIC values, restricted to only models with noise model None.

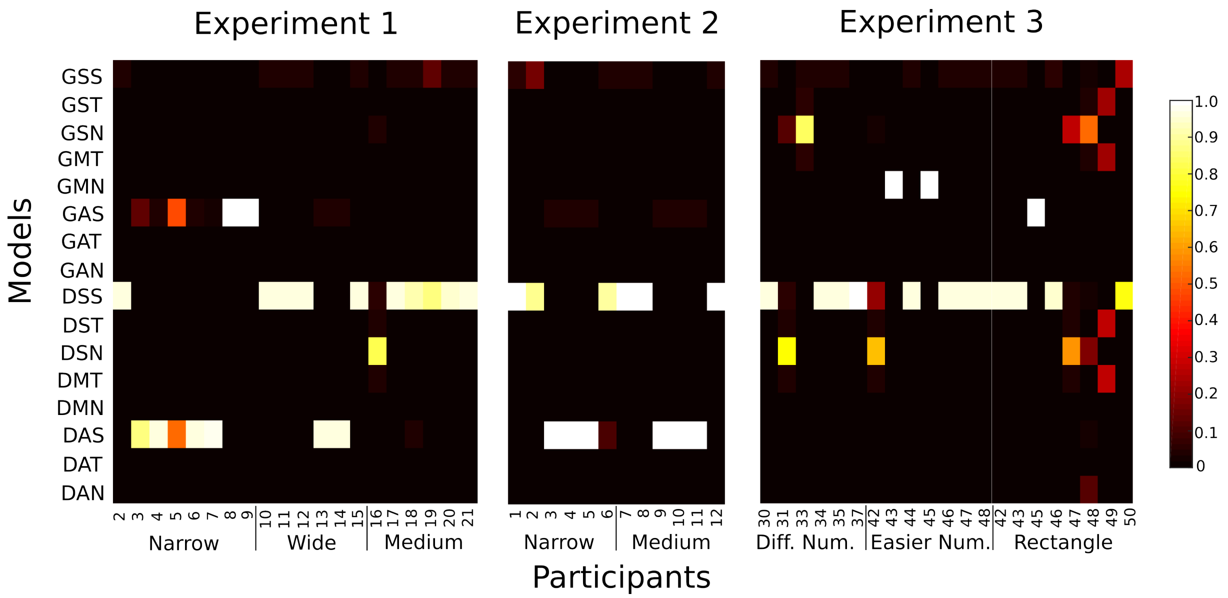

Figure 2. Heat map of the model posterior probabilities based on BIC values.

Table 4. True and fitted parameters for 15 simulated data sets.

Simulated Participant	True n	True psi	True beta	Best fit n	Best fit psi	Best fit beta
1	1	0.001	1	1	0.0005	1.0468
2	1	0.001	1	1	0.0004	0.9675
3	1	0.001	1	1	0.0025	1.0255
4	1	0.001	5	1	0.0027	6.4259
5	1	0.001	5	1	0.0069	3.4916
6	1	0.001	5	1	0.0034	4.1841
7	3	0.001	2.5	3	0.0052	2.5708
8	3	0.001	2.5	3	0.0038	2.186
9	3	0.001	2.5	3	0.0045	2.5117
10	1000	0.001	1	100	0.0006	1.0915
11	1000	0.001	1	1000	0.0004	0.9326
12	1000	0.001	1	1000	0.0026	0.9497
13	1	0.02	5	1	0.0175	3.8576
14	1	0.02	5	1000	0.038	49.9583
15	1	0.02	5	1	0.0217	5.3839

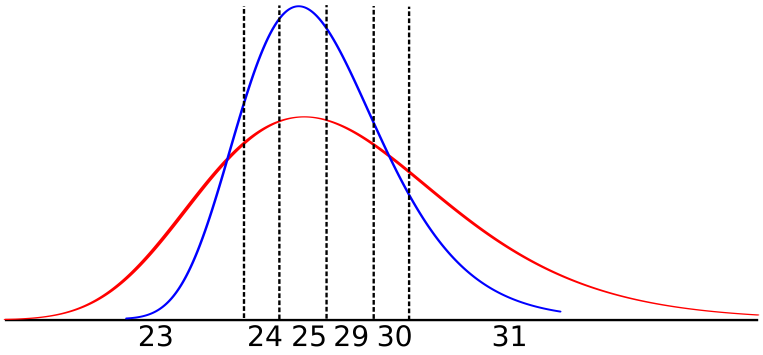

Figure 3. Diagram of how the distribution of discrete responses changes with likelihood width for a mapping. The blue and red curves are two lognormal distributions with different standard deviations. The dashed lines demarcate the different regions assigned to each number. The proportion of responses of each number corresponds to the area under a curve within a region.

A prior or a mapping?

This section addresses whether participants are actually learning a real prior distribution over the responses or whether they are learning a mapping: a set of learned boundaries that convert an impression of magnitude into a discrete response. In previous work, a linear in log-space mapping has formed the basis of an influential model of numerosity judgments [3]. This type of mapping cannot explain our results: linear mappings or monotonic transformations of linear mappings cannot contort themselves to produce multimodal distributions. The response distributions in this experiment can only be produced by a mapping that expands the proportion of responses to 23-25 and 29-31 at the expense of responses 26-28 and thus it is not a linear operation.

Previous research has tested a more general class of mappings with continuous responses by first training participants with a particular likelihood distribution and then observing how responses changed when they were tested with a wider likelihood distribution [4]. As shown in Equation 6 of the main text, if a prior is used then responses on average would be drawn more toward the prior when the width of the likelihood increased. However, for a mapping there is no additional feedback so the responses would not be drawn closer to the prior. In [4], the change in responses was consistent with a prior.

Unfortunately in discrete numerical estimation the results are not as simple to evaluate. To show why, an example of a mapping is shown in Figure 3. Here a single sample is taken from a lognormal distribution and the region into which the sample falls determines the response. As the width of the likelihood distribution increases more responses will be made to numbers 23 and 31, and as the width becomes very large nearly half of the responses will be made to each of these two numbers. Because the extreme responses are squashed into these numbers, the mapping can also predict that the mean response will be closer to the prior mean.

Instead of looking at the mean results when there is a wider likelihood distribution, we performed a quantitative model comparison. We compared the 'super-model' defined in the Materials and Methods section of the main text with a model that instantiates a mapping. Instead of using a prior distribution, the mapping model consisted of a series of thresholds that defined the boundaries between responses. As in Figure 3, the mass of the likelihood distribution falling into each region determined the probability of making that response. Like the 'super-model', the mapping model also used a fixed probability (0.001) of producing a uniform response over the range of responses from 1 to 100.

For each individual participant in Experiment 3, best-fitting parameters for both models were found for the first estimation block (500 trials) using the likelihood width estimated from a discrimination

block using the same display parameters. Then, using the likelihood width estimated from the other, more difficult discrimination block, both models made parameter-free predictions for the more difficult estimation block (200 trials). To prevent incorrect keypresses from influencing the results, responses less than 10 or greater than 100 were ignored in both the fitting and prediction stages. (This completely excluded the third participant of the difficult numerosity group, who made no responses greater than 10 in the more difficult estimation block.)

We compared the models in two ways. First we compared the two models predictions based for all numbers between 10 and 100 and found that thirteen of twenty participants were better fit by the prior distribution model than by the mapping model, most with large differences between the likelihoods of the predictions demonstrating a lot of heterogeneity in participant responses.

It is possible that the mapping model was predicting poorly because the training data did not constrain the parameters well – the parameters of the mapping model are more local and participants very rarely made responses outside of the presented range of values. A follow-up analysis grouped together all predictions and responses at each edge of the presented range with all values outside that range (i.e., 10-23 were grouped together and 29-100 were grouped together) and found that eleven of twenty participants were better fit by the prior model, further demonstrating evidence that a prior was used, but only evidence for some of the participants.

Some of this heterogeneity is potentially due to how the task was structured: because the training and test trials were separated, participants may not have carried over their prior from the first session to the second. In particular, this would explain the behavior of the participant who only produced numbers below ten in the second and more difficult estimation block.

Correlations between model parameters and response time

If participants are performing a form of approximate inference in which effort is traded off against accuracy, we might expect a correlation between the 'super-model' parameters and the average response time of participants. To test this, we correlated average response time with the number of samples n, the posterior distribution exponent β, and the Gaussian kernel width ψ both within experiment and across all experiments (Table 5). Participants with an average RT more than three standard deviations away from the mean across participants were excluded, removing one participant from Experiment 1.

We found no significant correlations between response time and any of the model parameters across any or all of the experiments ($p < .05$).

Table 5. Correlations between average response time and model parameters.

	n	β	ψ
E1 $\overline{\text{RT}}$	0.08	0.01	-0.02
E2 $\overline{\text{RT}}$	-0.35	-0.16	0.15
E3 $\overline{\text{RT}}$	-0.00	0.01	0.21
E1-3 $\overline{\text{RT}}$	-0.25	-0.12	0.18

References

1. Kass RE, Raftery AE (1995) Bayes factors. Journal of the American Statistical Association 90: 773-795.

2. Acerbi L, Ma W, Vijayakumar S (2014) A Framework for Testing Identifiability of Bayesian Models of Perception. In: Proc. Advances in Neural Information Processing Systems (NIPS 14). Montreal, Canada.

3. Izard V, Dehaene S (2008) Calibrating the mental number line. Cognition 106: 1221–1247.

4. Körding K, Wolpert DM (2004) Bayesian integration in sensorimotor learning. Nature 427: 244-247.
