# Supplementary material for: Fast and Accurate Learning When Making Discrete Numerical Estimates
Source: PLoS Comput Biol. 2016 Apr 12;12(4):e1004859. doi: 10.1371/journal.pcbi.1004859 (PMC4829178; doi:10.1371/journal.pcbi.1004859)
Supplement: S1 Dataset — (ZIP) [file pcbi.1004859.s002.zip › data_for_supporting_info/experiment_3/README.rtf]

In Experiment 3, the first and second discrimination sessions had different difficulty. Some participants second discrimination session files are labelled session 2 and some are labelled session 3, but both refer to the second session.
