# Supplementary material for: Fast and Accurate Learning When Making Discrete Numerical Estimates
Source: PLoS Comput Biol. 2016 Apr 12;12(4):e1004859. doi: 10.1371/journal.pcbi.1004859 (PMC4829178; doi:10.1371/journal.pcbi.1004859)
Supplement: S1 Dataset — (ZIP) [file pcbi.1004859.s002.zip › data_for_supporting_info/README.rtf]

Each folder contains raw data from a single experiment, with subfolders holding data from the different conditions within each experiment.Each file contains a number of variables, but the important one for data analysis purposes is exp_log. This contains the experimental data, while the practice trials are contained in ex_practice_log. Both of these structures have an entry for every trial arranged in the order in which it was shown to participants. The fields of exp_log depend on the task. In the discrimination sessions, participants tried to choose which of two screens contained more dots (or had a larger rectangle). The number of dots (or rectangle area) shown on the two trials were n_dots_anchor (or area_anchor) and n_dots_comparison (or area_comparison). The response field refers to the whether participants chose the first or second presented screen as containing the bigger number, and whether this was the anchor or comparison varied trial-by-trial according to the which_first field. The key response field is accuracy, which says whether a response was correct or not on a trial. In the estimation sessions, exp_log contains the numbers of dots (or rectangle area) shown in n_dots (or area) and the response made by participants in the response field. Accuracy is given in the accuracy field.
